# Histological and clinical phenotypes of diabetic kidney disease: a baseline analysis of the HEROIC study

**DOI:** 10.1093/ckj/sfag215

**Published:** 2026-06-25

**Authors:** Gordon G Paterson, Ortensia Vito, Lauren Heptinstall, Michael Sheaff, Abigail Lee, David Kim, Benjamin Challis, Robert Unwin, Magdi Yaqoob, Kieran McCafferty, Ben Caplin

**Affiliations:** Centre for Kidney and Bladder Health, Department of Renal Medicine, UCL, London, UK; Bart’s Health NHS Trust, London, UK; Translational Science and Clinical Development, Research and Early Development, Cardiovascular, Renal and Metabolism (CVRM), AstraZeneca BioPharmaceuticals R&D, Cambridge, UK; Department of Histopathology, Cambridge University Hospitals NHS Foundation Trust, Cambridge, UK; Bart’s Health NHS Trust, London, UK; Bart’s Health NHS Trust, London, UK; Genentech Inc, San Francisco, CA, USA; Translational Science and Clinical Development, Research and Early Development, Cardiovascular, Renal and Metabolism (CVRM), AstraZeneca BioPharmaceuticals R&D, Cambridge, UK; Centre for Kidney and Bladder Health, Department of Renal Medicine, UCL, London, UK; Translational Science and Clinical Development, Research and Early Development, Cardiovascular, Renal and Metabolism (CVRM), AstraZeneca BioPharmaceuticals R&D, Cambridge, UK; Bart’s Health NHS Trust, London, UK; William Harvey Research Institute, Queen Mary University of London, London, UK; Bart’s Health NHS Trust, London, UK; William Harvey Research Institute, Queen Mary University of London, London, UK; Centre for Kidney and Bladder Health, Department of Renal Medicine, UCL, London, UK; Royal Free London NHS Foundation Trust, London, UK

**Keywords:** CKD, CKD-EPI equation, diabetic kidney disease, GFR, kidney biopsy

## Abstract

**Background:**

Diabetic kidney disease (DKD) is increasingly recognized as a heterogeneous condition. Our understanding of the structure-function relationships underpinning DKD is limited. The East and North London Diabetes Cohort (HEROIC) study aims to identify prognostic markers in a diverse DKD cohort. We present here the baseline clinical phenotype of the cohort, define clusters of DKD and assess the performance of estimated GFR (eGFR) equations including CKD Epi 2021.

**Methods:**

Patients with diabetes and CKD were recruited from two tertiary renal units. Renal biopsies were assessed using the Renal Pathology Society scoring system. Ordinal logistic regression was used to assess clinical associations with histological severity and ensemble consensus clustering was used to identify distinct phenotypes. eGFR bias was calculated as measured GFR–eGFR.

**Results:**

In our cohort of 188 DKD patients (9.0% Black, 46.3% South Asian, 14.9% White, and 29.8% other ethnicity), there was an inverse relationship between BMI and glomerular lesion severity (OR 0.93, *P* = .008), which persists when restricted to type 2 diabetes patients. Ensemble consensus clustering identified five clusters with moderate separation including a phenotype with high rates of vascular disease, a phenotype with advanced DKD and low BMI, and one with a short duration of diabetes and milder glomerular lesions. CKD Epi 2021 overestimated eGFR in South Asian individuals whilst underestimation of eGFR was noted in Black individuals.

**Conclusions:**

We demonstrate clinically distinct clusters of DKD, which may provide hypotheses for ‘personalized’ approaches to interventions in DKD in future trials. We demonstrate that CKD-Epi 2021 performs poorly in a multi-ethnic DKD population.

## Graphical Abstract

**Graphical Abstract:**
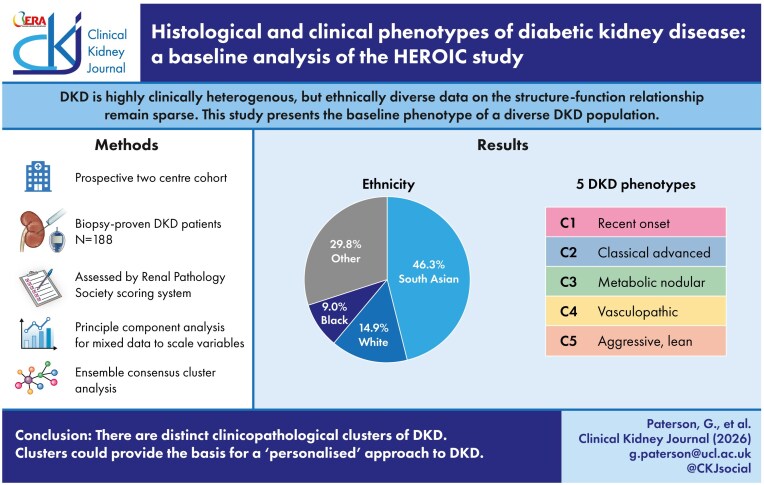

KEY LEARNING POINTS **What was known:**

- DKD is clinically heterogeneous but studies to date on structure-function relationships are primarily retrospective and have limited ethnic diversity.
- The performance of eGFR equations varies by population but the performance of CKD Epi 2021 has yet to be evaluated in a British cohort.

**This study adds:**

- We describe the baseline phenotype of a multi-ethnic, prospective cohort of biopsy-proven DKD.
- We identify distinct clinical phenotypes of DKD by cluster analysis in a diverse prospective study population.
- We assess the accuracy of CKD-Epi 2021 compared with measured GFR in a multi-ethnic British DKD cohort, revealing bidirectional ethnic bias with overestimation in South Asian and underestimation in Black individuals.

**Potential impact:**

- Clusters identified could provide the basis of ‘personalized’ treatment approaches in future trials.
- Clinicians ought to be aware of eGFR estimation errors with CKD-Epi 2021, particularly overestimation in South Asian individuals that may lead to undertreatment.

## INTRODUCTION

Diabetic kidney disease (DKD) is the leading cause of end-stage renal failure worldwide [1] and is routinely diagnosed without histological confirmation [2]. Biopsy is typically reserved for atypical cases defined by short uncomplicated history of diabetes, or suspicion of alternative pathology due to an active urinary sediment, excessive proteinuria or rapid GFR decline [3]. Histological examination, when performed, classically reveals thickened glomerular basement membranes, mesangial expansion and nodular sclerosis; these cardinal features are central to the Renal Pathological Society (RPS) framework for the assessment of DKD [4].

It is, however, increasingly appreciated that the histological phenotype of DKD is variable with some patients exhibiting predominantly non-classical lesions (e.g. vascular and interstitial lesions) [5, 6]. The clinical phenotype of DKD is similarly heterogeneous with variation in clinical features (e.g. albuminuria) and progression [7]. In other complex cardiometabolic diseases such as heart failure or type 2 diabetes (T2D) without renal complications, there is increasing interest in applying cluster analysis to identify distinct phenotypes [8–10], and to inform analyses of clinical trials [10–12].

Although several studies have examined the relationship between clinical features, biopsy findings, and renal outcomes [13, 14], the existing evidence base is limited to retrospective studies of clinically-indicated biopsies and thus may not be generalizable to the unselected DKD population [15]. Furthermore, there is a paucity of evidence from multi-ethnic cohorts with most studies originating from East Asia.

The East and North London Diabetes Cohort (HEROIC) Study is a two-centre, prospective cohort study of DKD which aimed to recruit from an ethnically diverse population [16], with follow-up due to finish later in 2026. Additionally, inclusion did not require a clinical indication for biopsy, allowing HEROIC to capture a broader spectrum of DKD. We report here the baseline cohort characteristics, identify predictors of lesion severity, utilize cluster analysis to explore clinical–histological associations and examine the relationship between measured and estimated GFR in this multi-ethnic DKD cohort, where such evidence remains limited.

## MATERIALS AND METHODS

### Patients

HEROIC recruited patients from Barts Health NHS Trust and Royal Free London NHS Foundation Trust, two tertiary renal centres in North Central and East London between 2019 and 2022. The protocol has been published previously [16]. Eligible participants were aged over 18, living with diabetes and CKD with an eGFR >30 ml/min/1.73 m². All participants were deemed to be at moderate or high risk of progression: moderate risk was defined as either a rapidly falling eGFR (>5 ml/min/1.73 m²/year decline in eGFR) or significant albuminuria (albumin:creatinine ration [ACR] >30 mg/mmol); high risk required both. Participation did not require a clinical indication for biopsy. Additional information can be found in supplementary methods.

### Clinical data synthesis

Baseline medical history including medications, medical history and demographic data were collected and clinical measurements were taken at a baseline visit. Weight category (healthy, overweight, obese) was assigned based on ethnicity-adjusted thresholds suggested by UK NICE overweight and obesity management guidelines (NG246) [17]. Additional information can be found in supplementary materials. Laboratory samples for this analysis were conducted in clinical laboratories. eGFR was calculated using the CKD-Epi 2021 formula [18] unless otherwise stated. Measured GFR (mGFR) was assessed by ^99m^Tc-DTPA plasma clearance. Non-alcoholic fatty liver disease was considered likely based on a NAFLD Ridge Score ≥ 0.44 [19].

Following informed consent patients underwent percutaneous renal biopsy. Histology was assessed by specialist renal histopathologists at one centre with consensus discussion for challenging cases. Patients with non-diabetic lesions requiring immunosuppression were excluded from the study follow-up and analysis. The RPS framework for DKD reporting was used to give semi-quantitative assessment glomerular lesions as well as non-glomerular vascular and interstitial lesions seen in DKD [4].

### Statistical analysis

To investigate predictors of histopathological lesions, 32 commonly assessed clinical, demographic, and laboratory variables were used. All variables were screened for association with RPS glomerular and IFTA scores by ordinal logistic regression adjusted for age, sex and ethnicity. Missing data was imputed using multivariate imputation by chained equations (MICE) [20] for ordinal logistic regression. Further information regarding imputation is available in supplementary methods.

We hypothesized that there may be clusters of patients with classic nodular sclerosis and other populations with alternative forms of glomerular pathology related to metabolic syndrome or vasculopathy. To test this, we performed a cluster analysis (excluding T1D patients). We selected commonly measured clinicopathological variables related to the hypothesis (measured at baseline): ACR, weight category, RPS glomerular score, number of antihypertensives, antiplatelet use (a proxy for atherosclerotic disease [21]), and retinopathy. To scale variables and reduce dimensionality we performed principal component analysis (PCA) of mixed data (PCAmixdata R package [22]). Ensemble consensus clustering was performed on the resulting principal components (PCs) which explained >60% of the variance [23]. For this analysis, since PCAmixdata is not currently compatible with MICE, missing data were imputed by multivariate imputation by factor analysis of mixed data (missMDA package [24]). Further details can be found in the supplementary methods.

To explore the performance of mGFR and eGFR equations each participant’s first mGFR was used. eGFR was calculated using creatinine-based equations: MDRD [25], CKD-EPI 2009 [26] (with and without race co-efficient), and CKD-Epi 2021 [18]. We evaluated the accuracy of estimating equations using bias (mGFR–eGFR) and the proportion within 30% of mGFR (P30). Confidence intervals for bias were derived directly from linear models; for other metrics they were calculated by bootstrapping with 1000 resamples (using boot package [27]). Additional information can be found in the supplementary methods.

Data analysis was conducted using R 4.5 [28].

### Sensitivity analyses

We undertook a sensitivity analysis without imputation. We also conducted ordinal logistic regression of prediction of RPS glomerular and IFTA score by clinical variables restricted to T2D patients.

### Ethics

Ethnical approval was given by London Bloomsbury Research Ethics Committee (19/LO/1921) and all participants gave informed consent.

## RESULTS

### Baseline cohort description

**Figure 1:**
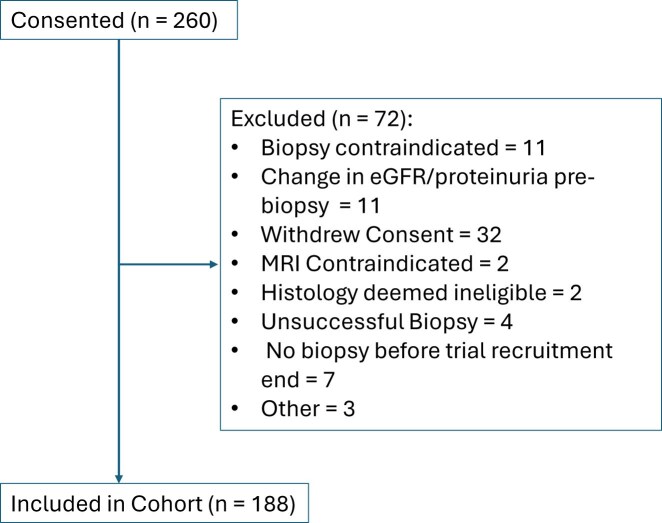
Recruitment diagram: consort diagram of patients consented, excluded and included in cohort.

A total of 188 patients were included in the study (Fig. 1) with a median age of 57 (IQR 49–62). The cohort is ethnically diverse: 9.0% Black, 46.3% South Asian, 14.9% White, and 29.8% other or mixed ethnicity. Most participants had T2D (170/188), with higher T1D rates in white participants (Table 1). Median eGFR at baseline was 47 ml/min/1.73 m^2^ (37–62.8). A total of 50.5% of the population met BMI obesity threshold and both rates of obesity and BMI varied between ethnicities with the lowest BMI 27.5 kg/m^2^ ( 24.7–31) seen in South Asian participants and the highest 31 kg/m^2^ (29.1–36.4) in Black participants (Table 1). Median ACR was 170.2 mg/mmol (77.3–281.3), and there was a small minimally-proteinuric population (18 participants ACR ≤30 mg/mmol of whom four had ACR ≤3 mg/mmol). Table 1 contains detailed clinical and demographic features stratified by ethnicity.

**Table 1:** Baseline clinical features by ethnicity.

Mean/median (SD/IQR) or % for count data							
Variable	Missing	Black	South Asian	White	Other	Whole cohort	*P*
Demographic features							
Age (yrs)^†^	0	59 (54, 60)	56 (47, 62)	57 (49, 60)	57 (50, 65)	57 (49, 62)	.84
Sex (% female)	0	35.3%	27.6%	21.4%	25%	26.6%	.763
Clinical Features							
BMI^†^	0	31 (29.1, 36.4)	27.5 (24.7, 31)	29.6 (25.6, 33.6)	27.6 (25.3, 32.3)	28 (25.3, 31.9)	0
SBP (mmHg)^†^	0	146 (138, 157)	141 (126, 160)	140 (127, 155)	143.5 (127, 156)	142 (127, 157)	.64
DBP (mmHg)^†^	0	84 (77, 89)	76 (68, 83)	78 (72, 86)	80 (74, 87)	78.5 (72, 85)	.01
HbA1c (mmol/mol)^†^	6	52.5 (46, 67)	65 (52, 79)	64 (52, 76)	59 (51, 70)	61 (52, 76)	.11
Diabetes Duration (yrs)^†^	0	8 (4.3, 17.7)	16.1 (7.9, 20.2)	14.9 (11, 24.1)	15.8 (7.5, 19.2)	15.4 (7.5, 20.2)	.21
ACR (mg/mmmol)^†^	20	134.1 (45.4, 200.8)	165.1 (81.2, 297.3)	200.1 (99.4, 335)	199.1 (69.2, 281.3)	170.2 (77.3, 281.3)	.4
eGFR (ml/min/1.73 m²)^†^	0	40.7 (36.2, 51.2)	48.7 (41.2, 66.3)	43.2 (34.1, 57.3)	45.9 (34.4, 62.3)	47 (37, 62.8)	.17
MAP (mmHg)^†^	0	104 (100.7, 110.7)	98.3 (90, 106.7)	99.5 (88.3, 106)	101.2 (93.7, 107.3)	100.7 (92.3, 107.3)	.05
Retinopathy (%)	0	70.6%	78.2%	64.3%	67.9%	72.3%	.396
T2D (%)	0	88.2%	97.7%	82.1%	83.9%	90.4%	.016
Obese (%)	0	88.2%	50.6%	50%	39.3%	50.5%	.006
Medications							
Insulin treated (%)	0	11.8%	24.1%	32.1%	28.6%	25.5%	.44
SGLT2i (%)	0	17.6%	43.7%	14.3%	30.4%	33%	.012
RAASi (%)	0	64.7%	72.4%	50%	66.1%	66.5%	.186
GLP-1 agonist (%)	0	5.9%	10.3%	3.6%	3.6%	6.9%	.38
RPS Glomerular Score							
I		23.5 %	11.5 %	10.7 %	8.9 %	11.7 %	
IIa		29.4 %	24.1 %	17.9 %	21.4 %	22.9 %	
IIb		5.9 %	21.8 %	17.9 %	23.2 %	20.2 %	
III		35.3 %	26.4 %	46.4 %	35.7 %	33 %	
IV	2	0 %	14.9 %	7.1 %	10.7 %	11.2 %	.36
RPS IFTA Score							
0		0 %	1.1 %	0 %	0 %	0.5 %	
1		35.3 %	48.3 %	42.9 %	50 %	46.8 %	
2		47.1 %	35.6 %	53.6 %	37.5 %	39.9 %	
3	0	17.6 %	14.9 %	3.6 %	12.5 %	12.8 %	.752
RPS Arteriolar Hyalinosis Score							
0		11.8 %	4.6 %	7.1 %	8.9 %	6.9 %	
1		35.3 %	23 %	17.9 %	28.6 %	25 %	
2	0	52.9 %	72.4 %	75 %	62.5 %	68.1 %	.251
RPS Interstitial Inflammation Score							
0		11.8 %	17.2 %	3.6 %	12.5 %	13.3 %	
1		88.2 %	74.7 %	82.1 %	83.9 %	79.8 %	
2	0	0 %	8 %	14.3 %	3.6 %	6.9 %	.185

Data were collected at initial visits. Data are presented as mean (SD) if normally distributed (‡) or median (IQR) if not normally distributed (†). Count data are presented as percentage. Comparisons were made using ANOVA or Kruskall-Wallis based on normality.

IMD, index multiple deprivation.

There was a high prevalence of coded hypertension (77% of participants) at baseline. Coronary artery disease was present in 9.6% of participants; cerebrovascular disease in 9.6% and heart failure in 7.4%. The median 5-year Kidney Failure Risk Equation (KFRE) score was 3.4% (0.7%–11.1%, Supplementary Table S1).

There was a range of glomerular pathology observed. The most common RPS glomerular grade was III (presence of nodular sclerosis) seen in 33% of participants with over half of participants having less severe lesions (Table 1). Similarly, there was a spread of IFTA scores between 1, 2, and 3 (representing 0–25%, 25%–50%, >50% of the interstitium, respectively). In contrast, there was limited variability in the vascular lesion and interstitial inflammation scores (Table 1). Two participants had no glomerular score available or no abnormality and were excluded from subsequent analyses.

### Association of clinical variables with histological lesion severity

We sought to explore the clinical and demographic predictors of RPS glomerular scores. Distributions of imputed data are available in supplementary sensitivity analyses. Ten variables were significant predictors of glomerular score after adjustment for age, sex, and ethnicity (Supplementary Table S2). The strongest predictors of glomerular class were retinopathy and insulin treatment. Increasing BMI was associated with lower glomerular scores (OR 0.93, 95% CI 0.88–0.98), as was increasing weight category (OR 0.56, 95% CI 0.32–0.99). In sensitivity analysis limited to patients with T2D, the direction of associations remained unchanged (supplementary sensitivity analyses). Increasing IFTA score was associated with 10 clinical variables (Supplementary Table S3).

### Ensemble consensus clustering

**Figure 2:**
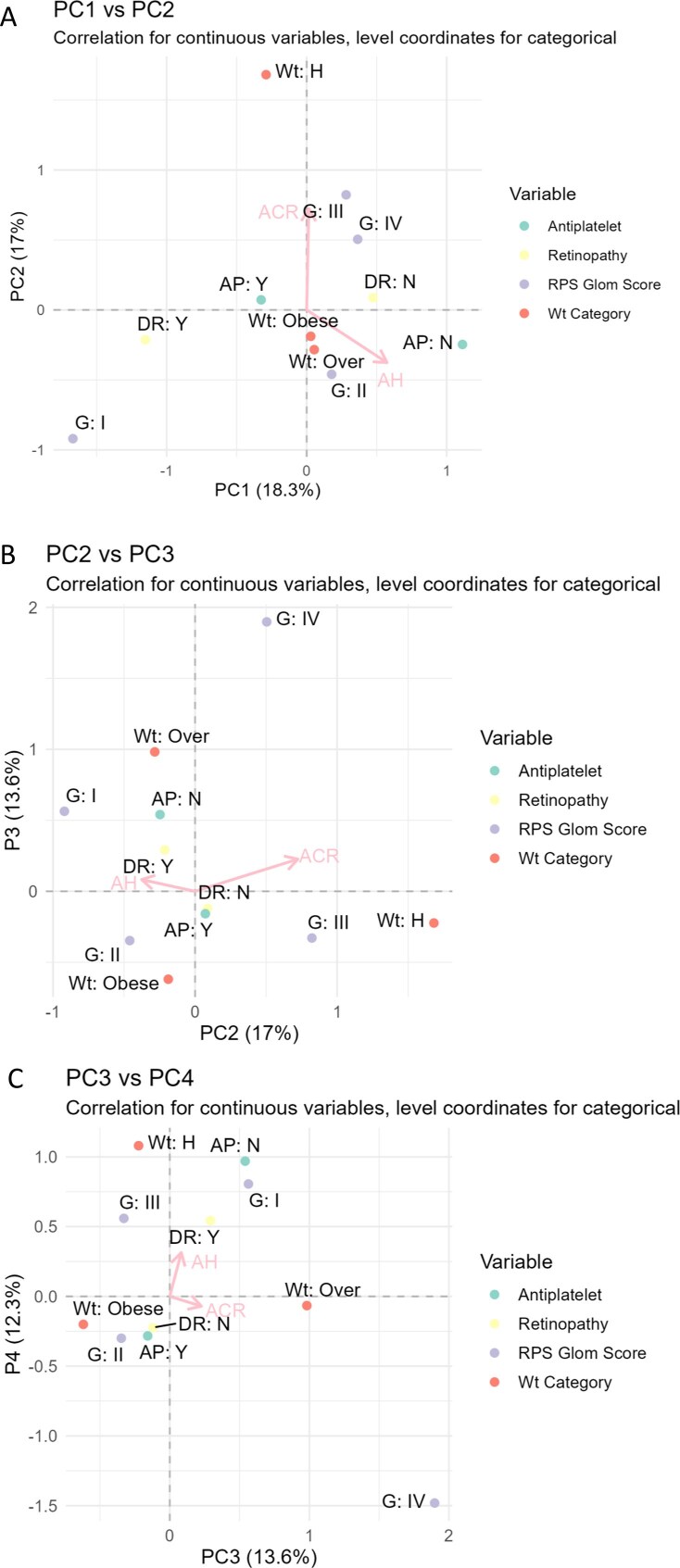
PCA for mixed data was performed on selected variables. The level co-ordinates for categorical variables are shown with correlation vectors of continuous traits overlaid for PC1 vs PC2 (A), PC2 vs PC3 (B), PC3 vs PC4 (C). G, glomerular score; AH, anti-hypertensives; AP, antiplatelet use; DR, retinopathy; Wt: H, healthy weight; Wt: Over, overweight; Wt: Obese, obese.

Given the regression associations, we hypothesized that there may be clinical characteristics associated with classical glomerular changes and others with less advanced glomerular features. We therefore performed a cluster analysis to look for distinct phenotypic clusters. PCA showed four PCs explained 61.2% of the variation. Variable contributions to PCs are shown in Fig. 2. Ensemble consensus clustering identified moderate clustering (silhouette score 0.34, indicating partial phenotypic overlap) with 5 clusters labelled descriptively as:

- Cluster 1 (*n* = 30): *Recent-onset*—lowest ACR, moderate obesity rates, short duration of diabetes, little retinopathy, mild histological changes.
- Cluster 2 (*n* = 15): *Classical advanced*—second highest ACR, second lowest obesity rates, long duration of diabetes, high rates of retinopathy, advanced glomerular changes.
- Cluster 3 (*n* = 71): *Metabolic nodular*—second lowest ACR, highest obesity rates, long duration of diabetes, high retinopathy prevalence, nodular sclerosis, and global glomerulosclerosis.
- Cluster 4 (*n* = 35): *Vasculopathic*—high rates of vascular co-morbidity, moderate ACR, moderate obesity rates, long duration of diabetes, high prevalence of retinopathy, mix of nodular sclerosis, and mesangial expansion.
- Cluster 5 (*n* = 17): *Aggressive, lean*—highest ACR (nephrotic), lowest BMI and obesity rates, long duration of diabetes, moderate retinopathy rates, high HbA1c, primarily nodular sclerosis.

**Figure 3:**
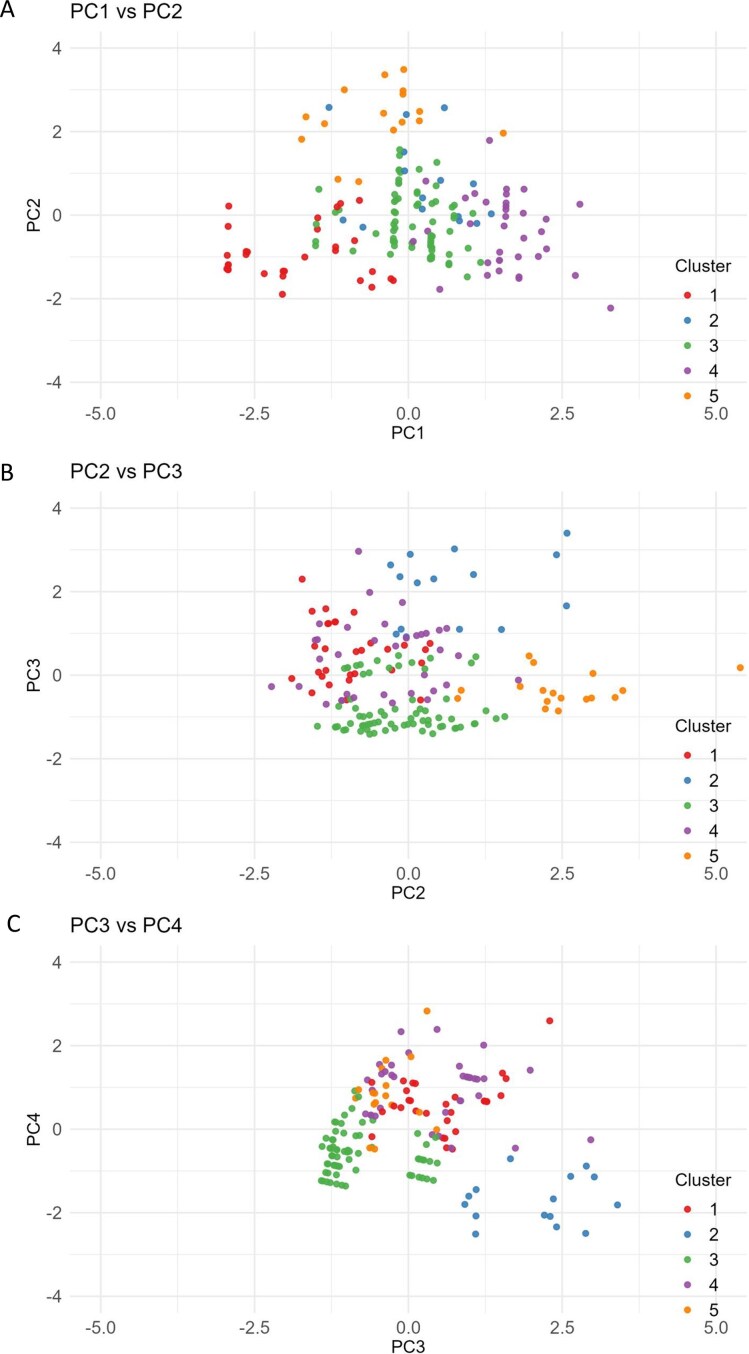
Ensemble clustering was performed on the 4 principal components (PCs) which explained >60% of variation. A, B and C show the individual coordinates for each participant in PC1 vs PC2, PC2 vs PC3, PC3 vs PC4 respectively, with cluster assignment depicted by colour.

Individual coordinates in PC spaces are shown in Fig. 3 and median PC scores for each cluster are shown in Supplementary Figure S1. Selected differences between clusters are highlighted in Table 2 (further variables in Supplementary Table S4). Imputation distributions, and analysis without imputed cases are available in supplementary sensitivity analysis.

**Table 2:** Comparison of clusters.

Variable	C1: Recent onset	C2: Classical advanced	C3: Metabolic nodular	C4: Vasculopathic	C5: Advanced, lean	*P*
n ()	30	15	71	35	17	
Clinicodemographic data						
ACR (mg/mmmol)	117.353	197.895	127.353	173.043	412.431	.00
Age (yrs)	52	52	58	60	59	.01
Antihypertensives (no. of agents)	1	1	1	3	1	.00
Antiplatelet (%)	6.667	13.333	1.408	88.571	11.765	.00
BMI (kg/m^2^)	26.823	26.019	31.038	27.616	23.343	.00
CAD (%)	6.667	0	2.817	31.429	5.882	.00
CVA (%)	10	20	7.042	20	0	.11
Diabetes duration (yrs)	4.717	16.011	15.272	17.621	11.619	.00
HbA1c (mmol/mol)	52	54	63	66.5	78	.01
Insulin (%)	3.333	33.333	19.718	34.286	17.647	.03
Obese (%)	40	33.333	74.648	40	23.529	.00
Retinopathy (%)	10	80	87.324	91.429	58.824	.00
Sex (% female)	33.333	33.333	26.761	14.286	29.412	.43
eGFR (ml/min/1.73 m²)	53.804	44.551	44.818	45.947	54.15	.18
RPS pathology scores						
Art Hyalinosis 0	8 (26.7%)	1 (6.7%)	1 (1.4%)	3 (8.6%)	0	
Art Hyalinosis 1	14 (46.7%)	3 (20%)	21 (29.6%)	6 (17.1%)	1 (5.9%)	
Art Hyalinosis 2	8 (26.7%)	11 (73.3%)	49 (69%)	26 (74.3%)	16 (94.1%)	.00
Glomerular I	20 (66.7%)	0	0	1 (2.9%)	0	
Glomerular II	8 (26.7%)	0	48 (67.6%)	16 (45.7%)	3 (17.6%)	
Glomerular III	2 (6.7%)	0	23 (32.4%)	16 (45.7%)	14 (82.4%)	
Glomerular IV	0	15 (100%)	0	2 (5.7%)	0	.00
IFTA 0	0	0	0	1 (2.9%)	0	
IFTA 1	20 (66.7%)	0	35 (49.3%)	18 (51.4%)	7 (41.2%)	
IFTA 2	10 (33.3%)	9 (60%)	28 (39.4%)	11 (31.4%)	7 (41.2%)	
IFTA 3	0	6 (40%)	8 (11.3%)	5 (14.3%)	3 (17.6%)	.00
Ethnicity						
Black (%)	13.333	0	14.085	0	0	
Other (%)	26.667	26.667	32.394	20	29.412	
South Asian (%)	46.667	66.667	43.662	62.857	41.176	
White (%)	13.333	6.667	9.859	17.143	29.412	.04

Data is presented as median or count data for categorical variables. Comparisons were made using ANOVA or Kruskall-Wallis based on normality. Count data is presented as percentage with comparisons by Chi-Squared test. For RPS classification *P*-values represent Kruskall-Wallis testing for the parameter.

### Validation of eGFR equations in a multi-ethnic DKD cohort

Since there is limited data on eGFR equation performance in multi-ethnic British cohorts, we also explored the performance of eGFR equations in our cohort. All three eGFR equations had suboptimal performance when compared to mGFR with P30 varying from 68.9% (61.6, 76.2) with CKD-Epi 2021 to 75.6% (68.3, 82.3) with MDRD (Supplementary Table S5). Given the widespread adoption of CKD-Epi 2021 across the UK and its poor performance in our analysis, we sought to determine if its performance varied by ethnicity. CKD-Epi 2021 demonstrated bidirectional ethnic bias: significant underestimation in Black participants (bias +7.6 ml/min/1.73 m², 95% CI 2.3–13.1) versus significant overestimation in South Asian participants (bias −7.5 ml/min/1.73 m², 95% CI −10.1 to −4.8, Supplementary Table S6).

## DISCUSSION

We present here the baseline clinical and demographic data of the HEROIC cohort. This work offers three main contributions: we describe an ethnically diverse cohort with DKD, describe five clinicopathological phenotypes of DKD and, concerningly, reveal that CKD-Epi 2021 shows significant and bidirectional ethnic bias in a multi-ethnic British DKD cohort.

### Baseline cohort description

HEROIC has successfully recruited a multi-ethnic cohort: UK Diabetes Audit 2024 data for North East London shows a prevalent T2D population which is 49% British Asian; 17% Black; 28% White [29] which HEROIC has broadly matched. The population is also clinically heterogenous: we report a wide range of histological features and renal function (both in terms of eGFR and proteinuria).

Compared with the US-based TRIDENT study [30], HEROIC participants are at an earlier stage of DKD, a group which has not been historically well studied. HEROIC participants are less proteinuric (PCR 214 mg/mmol vs 524 mg/mmol) with a higher baseline eGFR (mean 54 vs 32 ml/min/1.73 m²) [30]. Whilst HEROIC appears to have significant similarities to the BEAt-DKD cohort study [31], comparison between our studies at present is not possible given the baseline characteristics of the BEAt-DKD cohort have not yet been published.

Although we recruited a less proteinuric population than comparable studies and our inclusion criteria intended to allow the participation of rapidly progressing but non-proteinuric patients, we have only recruited a small minimally-proteinuric population. The median 5-year KFRE of 3.4%, however, confirms recruitment of a lower-risk population. Furthermore, our cohort has considerably lower rates of cardiovascular disease than previous cohort studies: the prevalence of ischaemic heart disease in our population was only ∼10%, whilst in the chronic renal insufficiency study’s diabetes cohort, this figure was 29% [32].

A similar pattern is seen when comparing histology: the HEROIC cohort had lower RPS glomerular and IFTA scores compared with TRIDENT [30]. This likely reflects our recruitment: TRIDENT recruited from patients undergoing clinically indicated biopsies, whilst HEROIC performed some biopsies which would be considered primarily research biopsies, not necessarily standard of care clinically.

### Associations between clinical and histological features

We sought to investigate which clinicodemographic features could predict RPS Glomerular and IFTA scores. The inverse relationship between BMI and glomerular class was unexpected and requires careful interpretation and could represent differences in adherence/risk factor control, or the presence of distinct phenotypes. We postulated that the impact of BMI on glomerular class may be related to groups of patients with different clinical characteristics, all with a histological diagnosis of DKD. The five phenotypic clusters identified highlight the heterogeneity of DKD beyond traditional staging.

The mild glomerular changes and short duration of diabetes of cluster 1 (*Recent-onset metabolic)* challenge traditional association between DKD severity and hyperglycaemia and support the role of non-glycaemic factors in the development of DKD [33].

Although cluster 2 (*Classical advanced*) and cluster 3 (*Metabolic nodular*) are statistically distinct, they share several clinical traits, including a long duration of diabetes, high prevalence of retinopathy, and relatively low rates of vascular disease. They vary primarily in relation to BMI and obesity with cluster 3 being the cluster with the highest rates of obesity.

Cluster 4 (*Vasculopathic*) in contrast is defined by vasculopathy and advanced diabetes, but this is not reflected in the RPS arteriosclerosis score. This may be because significant renovascular lesions occur more proximally (e.g. in the renal artery) or because the semi-quantitative RPS arteriosclerosis scale is not sensitive enough to detect differences between clusters.

Cluster 5 (*Aggressive lean*) represents aggressive disease with severe proteinuria and low median BMI. This phenotype is consistent with relative insulin deficiency due to pancreatic β-cell depletion, leading to aggressive nephropathy driven by sustained hyperglycaemia. The combination of low BMI and severe histological injury is consistent with the inverse BMI-glomerular class association and supports a pathophysiology distinct from obesity-associated kidney disease.

Previous clustering of CKD patients revealed a DKD cluster characterized by high rates of cardiovascular complications, similar to our cluster 4 [34]. Montero *et al*. described an advanced DKD cluster broadly analogous to our Cluster 2 [34]; however, the higher eGFR observed in our cohort likely reflects our inclusion criterion of eGFR >30 ml/min/1.73 m².

Identifying novel clusters could provide insights into mechanisms behind the heterogeneity of DKD. Medical therapy in DKD has expanded substantially over the last decade with nsMRA, GLP-1 agonists, and SGLT2 inhibitors, yet optimal timing and combinations remain unclear [35]. Phenotypic clusters could be used to as the basis for studies of optimal treatment combinations or sequencing.

### Implications of eGFR inaccuracy in a multi-ethnic cohort

To our knowledge, we are among the first to compare mGFR to CKD-Epi 2021 in the UK population and our results are concerning. Of note, the P30 reported here is significantly lower than those reported in equation derivation cohorts: our P30 for CKD Epi 2009 and 2021, respectively were 68.9% and 72.6%, whilst in the CKD Epi 2021 development cohort these equations had P30s above 85% [18].

CKD-Epi 2021 demonstrated significant bidirectional ethnic bias: overestimation in South Asian individuals (bias −7.5 ml/min/1.73 m²) and underestimation in Black individuals (bias +7.6 ml/min/1.73 m²). These biases may be clinically significant: underestimation of eGFR in Black individuals may result in over-treatment while eGFR overestimation in South Asian individuals is particularly concerning as it may delay treatment in a population with poor outcomes in DKD [36]. Further studies are needed to determine whether alternative measurements of renal function, such as cystatin C-based equations, perform better in these populations.

## LIMITATIONS

This work has several limitations. Without longitudinal data, the clinical relevance of our clustering is uncertain: future work examining the association between these clusters and cardiovascular and renal outcomes is warranted to validate these findings. Furthermore, this study is of moderate size and lacks a replication cohort. The association between obesity and glomerular class relies on BMI as a marker of obesity, which is a suboptimal marker of adiposity, and future work should consider validating this finding with body composition metrics such as waist circumference or bioimpedance. Our clusters are only of moderate strength (silhouette score 0.34), indicating some phenotypic overlap; the five-cluster solution is exploratory and may not replicate in external cohorts, and as a hypothesis-generating analysis none of the estimates were adjusted for multiple testing. Additionally, we currently only have semi-quantitative RPS scores, and future work with whole slide imaging, and detailed feature recognition may yield greater insights.

Cystatin C-based eGFR equations were not evaluated, which limits the completeness of our GFR estimation analysis, particularly given the ethnic bias observed with creatinine-based equations. Ethnicity-stratified eGFR analyses had limited statistical power, especially for the Black population, as reflected by wide confidence intervals. Finally, due to the covid pandemic visit intervals fell outside protocol and some study visits were missed completely and methods used to account for this may have introduced some bias.

## CONCLUSION

We present here baseline data on a highly phenotyped, diverse population living with biopsy confirmed DKD. Concerningly, we have shown that commonly used eGFR equations perform poorly in a multi-ethnic British DKD population, with bidirectional ethnic bias that has important implications for clinical care. We demonstrate varied RPS scores driven by clusters of patients with divergent clinical features, including one cluster with short duration of diabetes and mild DKD lesions and an advanced DKD cohort with lower BMI. This type of clustering may provide the basis for testing a ‘personalized’ approach to interventions in DKD in clinical trials.

## Supplementary Material

sfag215_Supplemental_Files

## Data Availability

The data underlying this article will be shared on reasonable request to the corresponding author.
