# Supplementary material for: Histological and clinical phenotypes of diabetic kidney disease: a baseline analysis of the HEROIC study
Source: Clin Kidney J. 2026 Jun 25;19(7):sfag215. doi: 10.1093/ckj/sfag215 (PMC13373966; doi:10.1093/ckj/sfag215)
Supplement: sfag215_Supplemental_Files [file sfag215_supplemental_files.zip › Sensitivity_Analyses_V3.docx]

***Sensitivity Analyses***

*Distribution of imputed datasets*


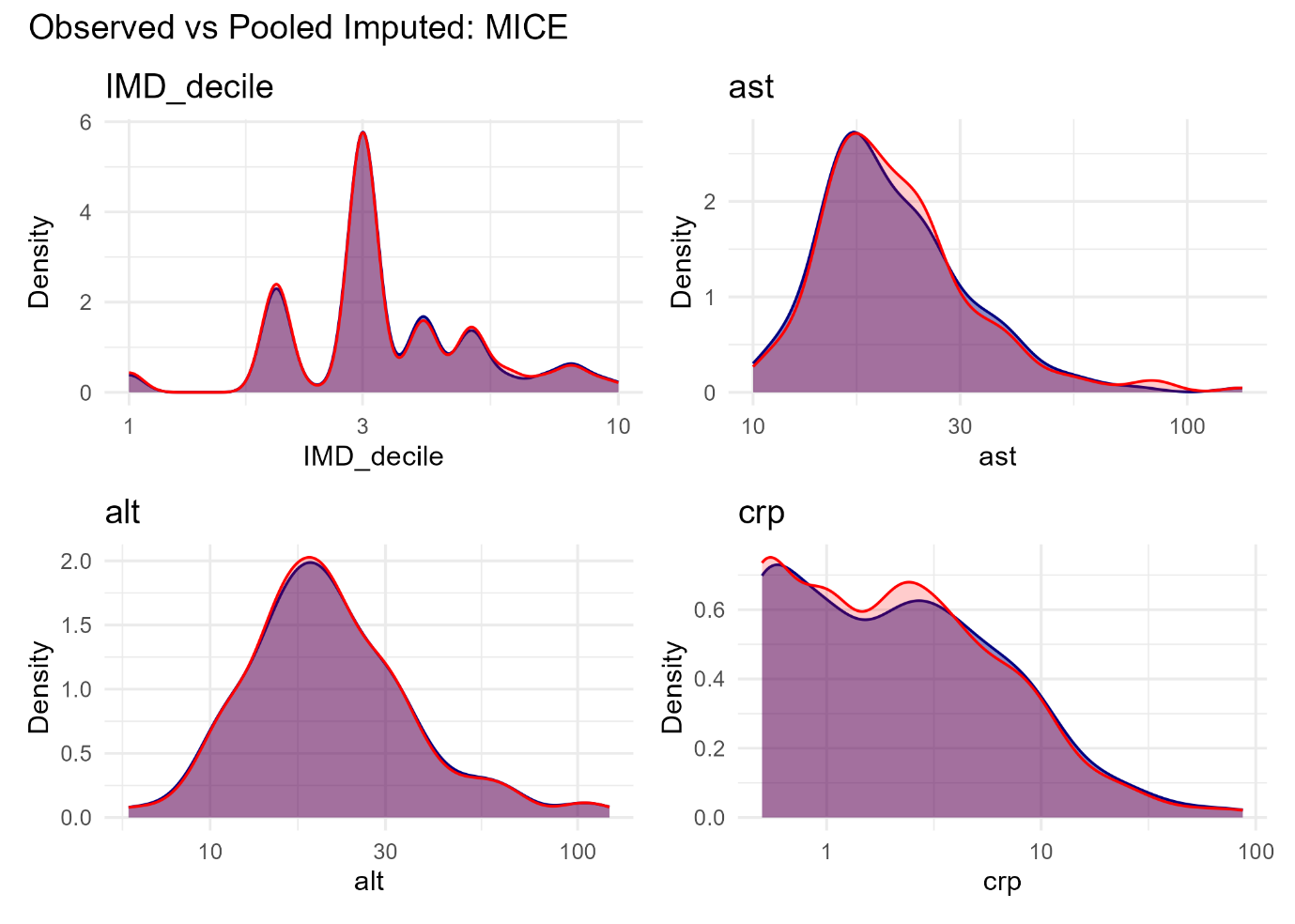
After imputation, we compared the distribution of imputed data vs observed data. There were minimal differences between the impmputed and observed distribution.


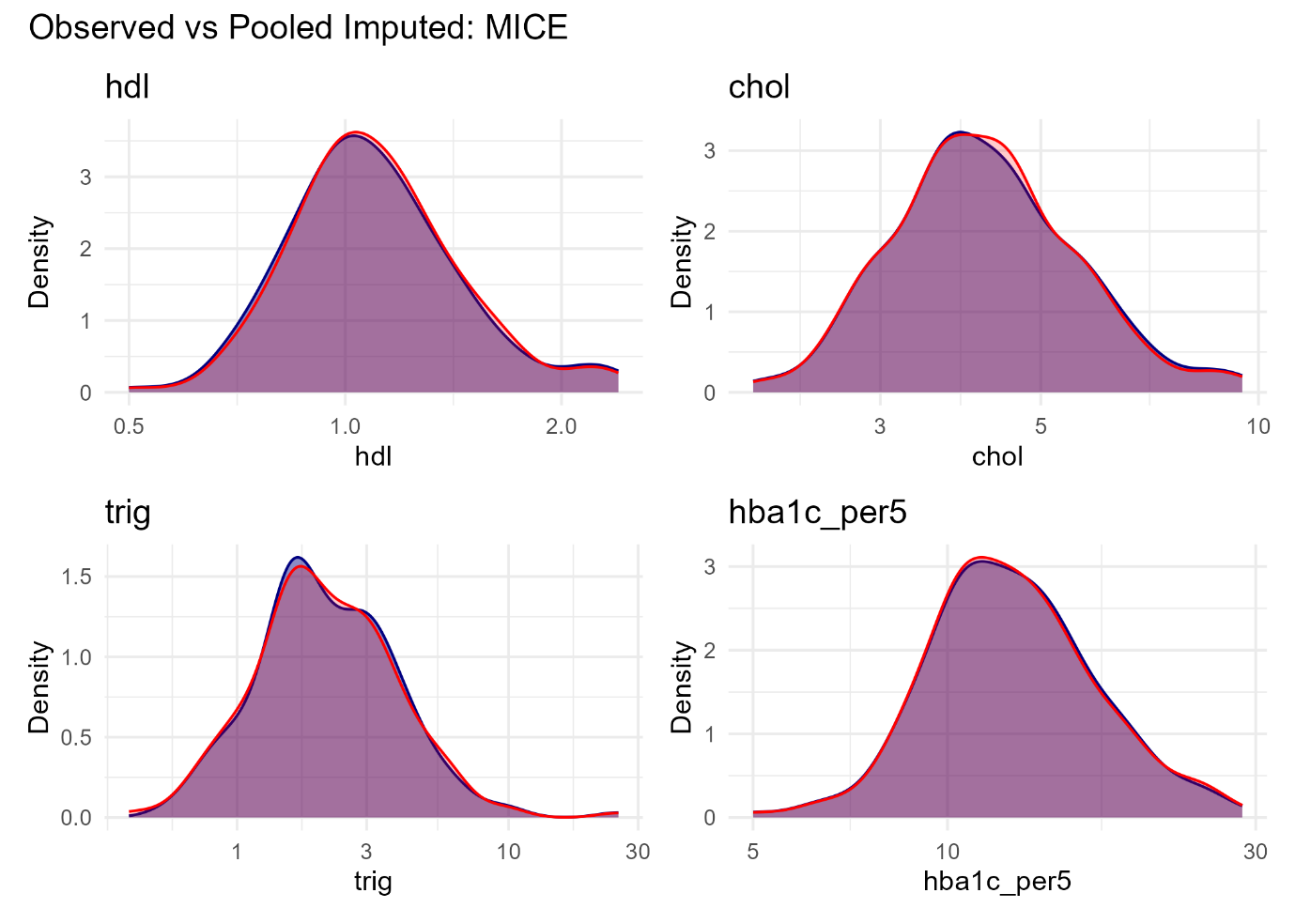

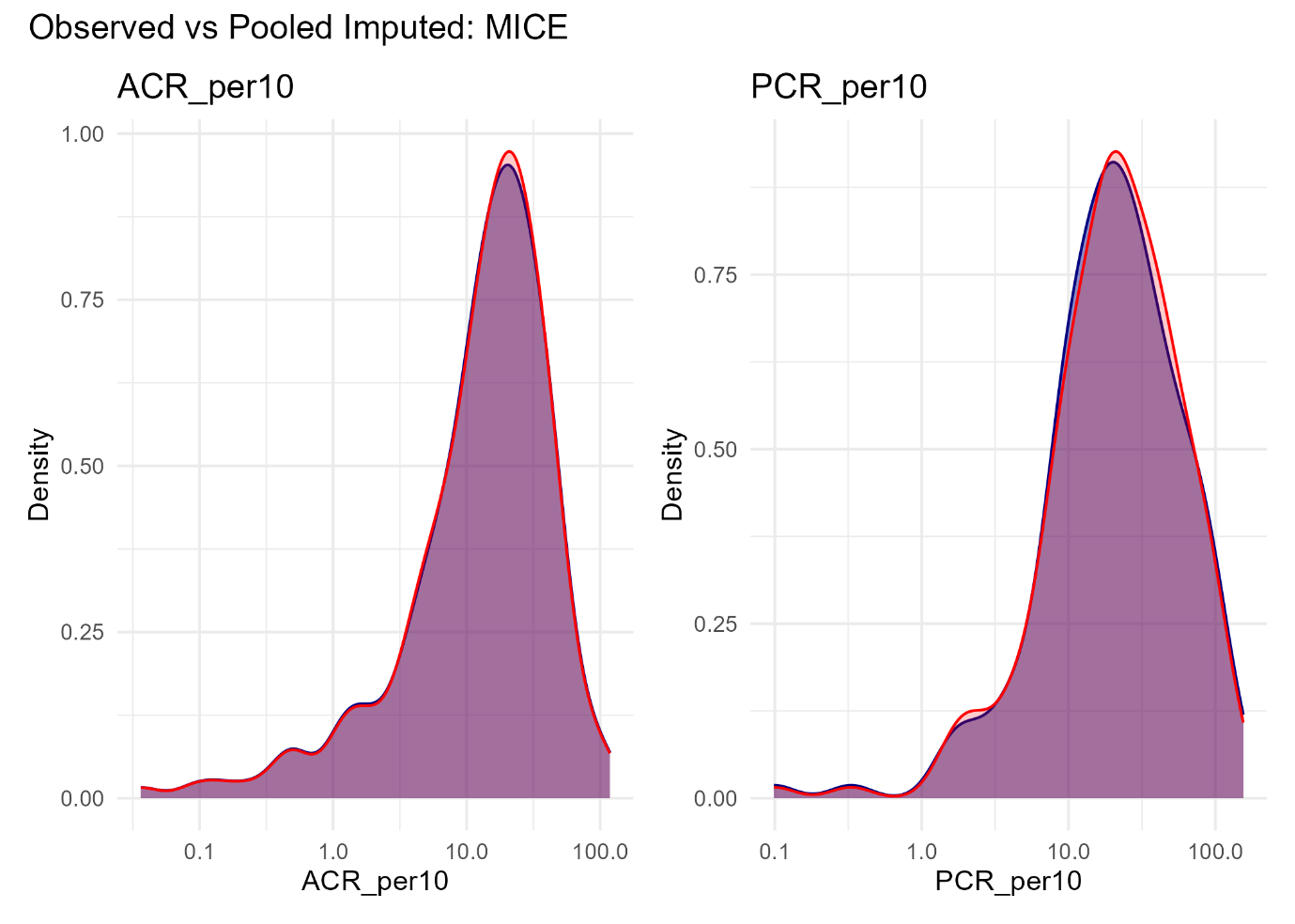


Observed vs Completed (with imputation) distributions for MICE. Continuous variables employed as predictors in ordinal linear regression with missing data underwent imputation by MICE. The observed data distribution is presented in navy. The mean completed data (over m = 10 imputations) is presented in red.


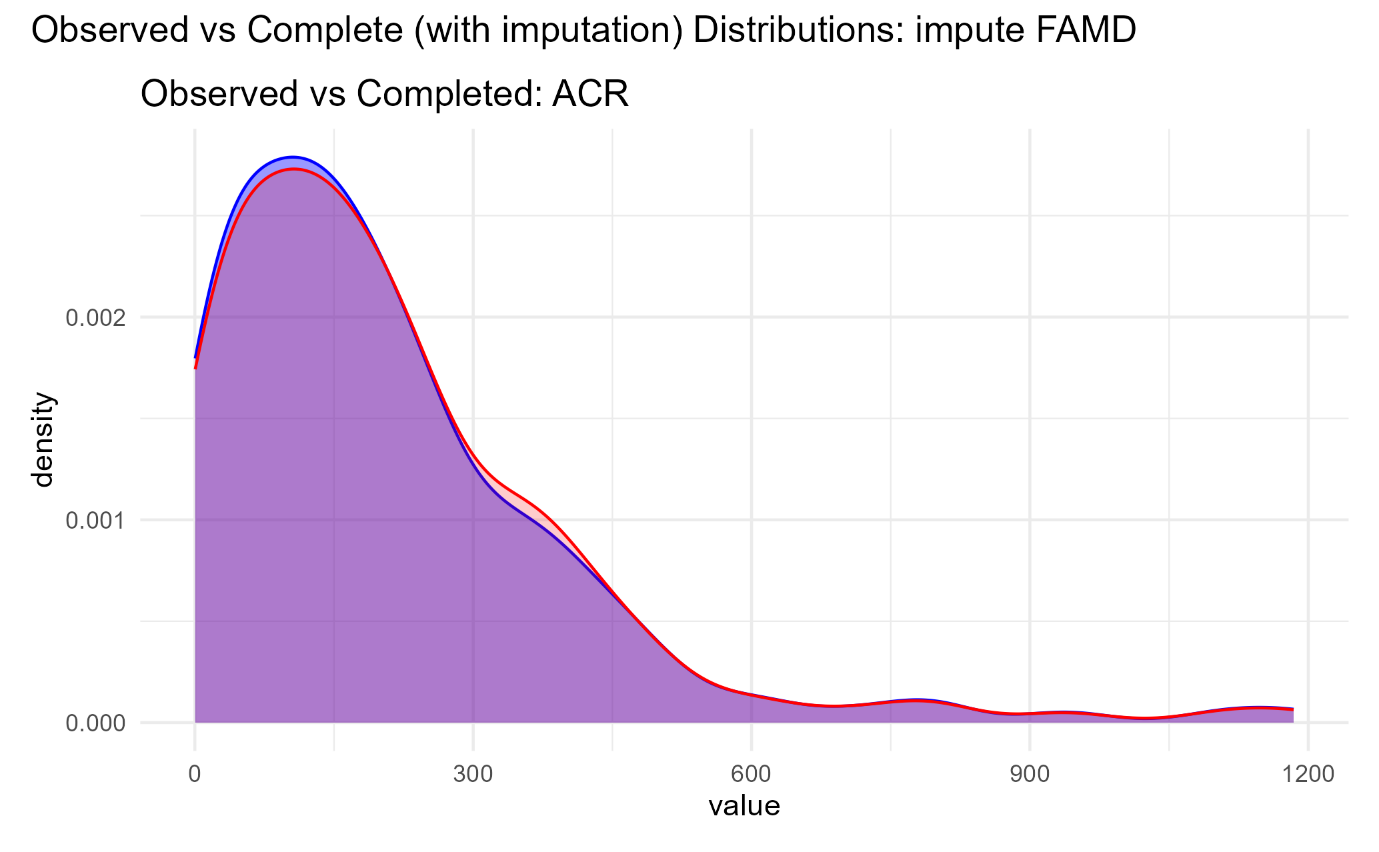
Observed vs Completed (with imputation) distributions by imputeFAMD. Continuous variables in our cluster analysis with missing data underwent imputation by imputeFAMD. The observed data distribution is presented in navy. The completed data (with imputation) is presented in red.

*Analyses without T1D: Ordinal Logistic Regression*

The association of glomerular scores with clinical variables was repeated excluding T1D cases.


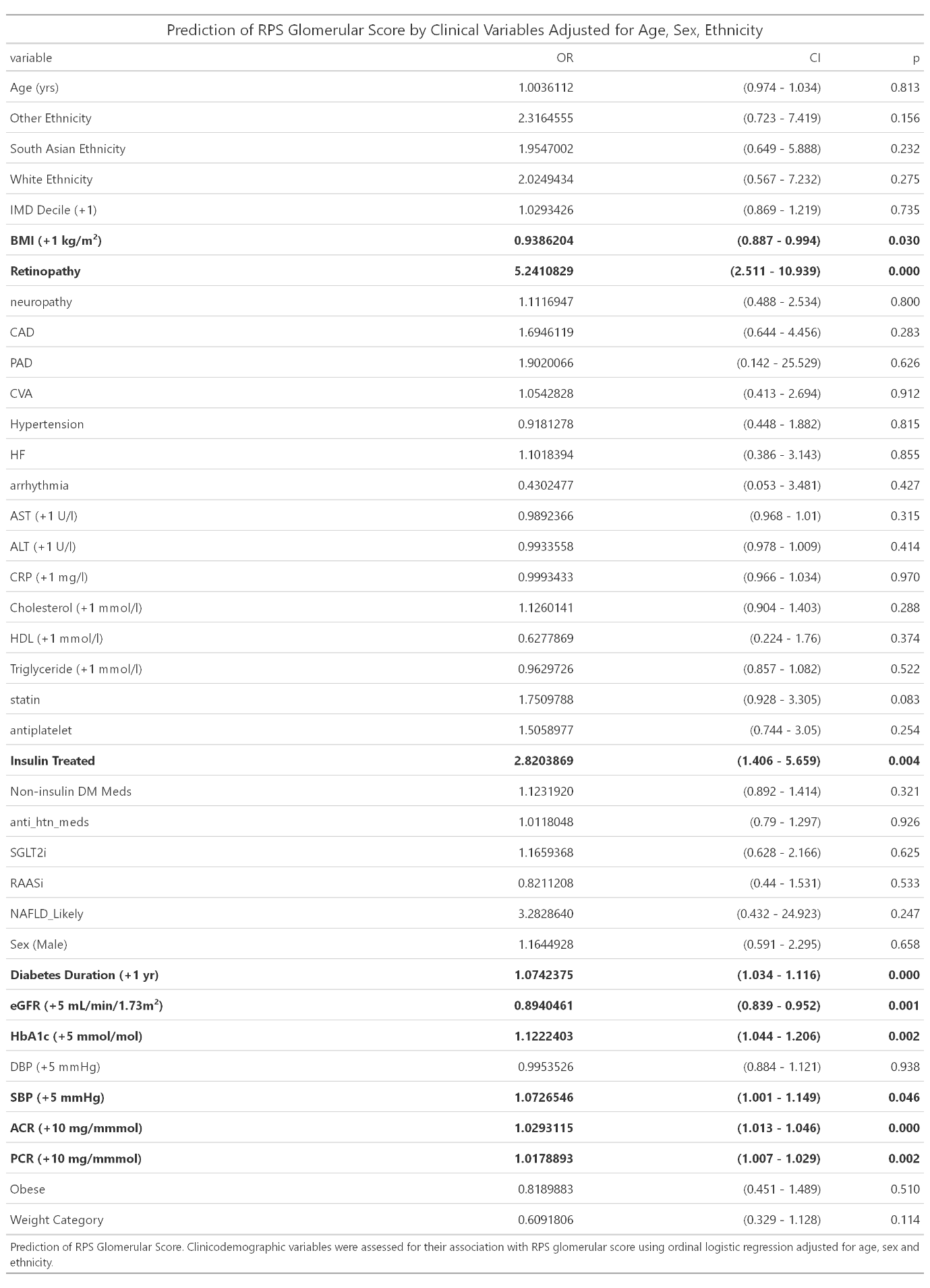


Age, Sex and Ethnicity Adjusted Prediction of RPS Glomerular Score by Clinical Variables, excluding T1D cases. Clinicodemographic variables were assessed for their association with RPS glomerular score using ordinal logistic regression adjusted for age, sex and ethnicity.

*Analyses without Imputation: Ordinal Logistic Regression*

Ordinal logistic regression was performed on completed cases.


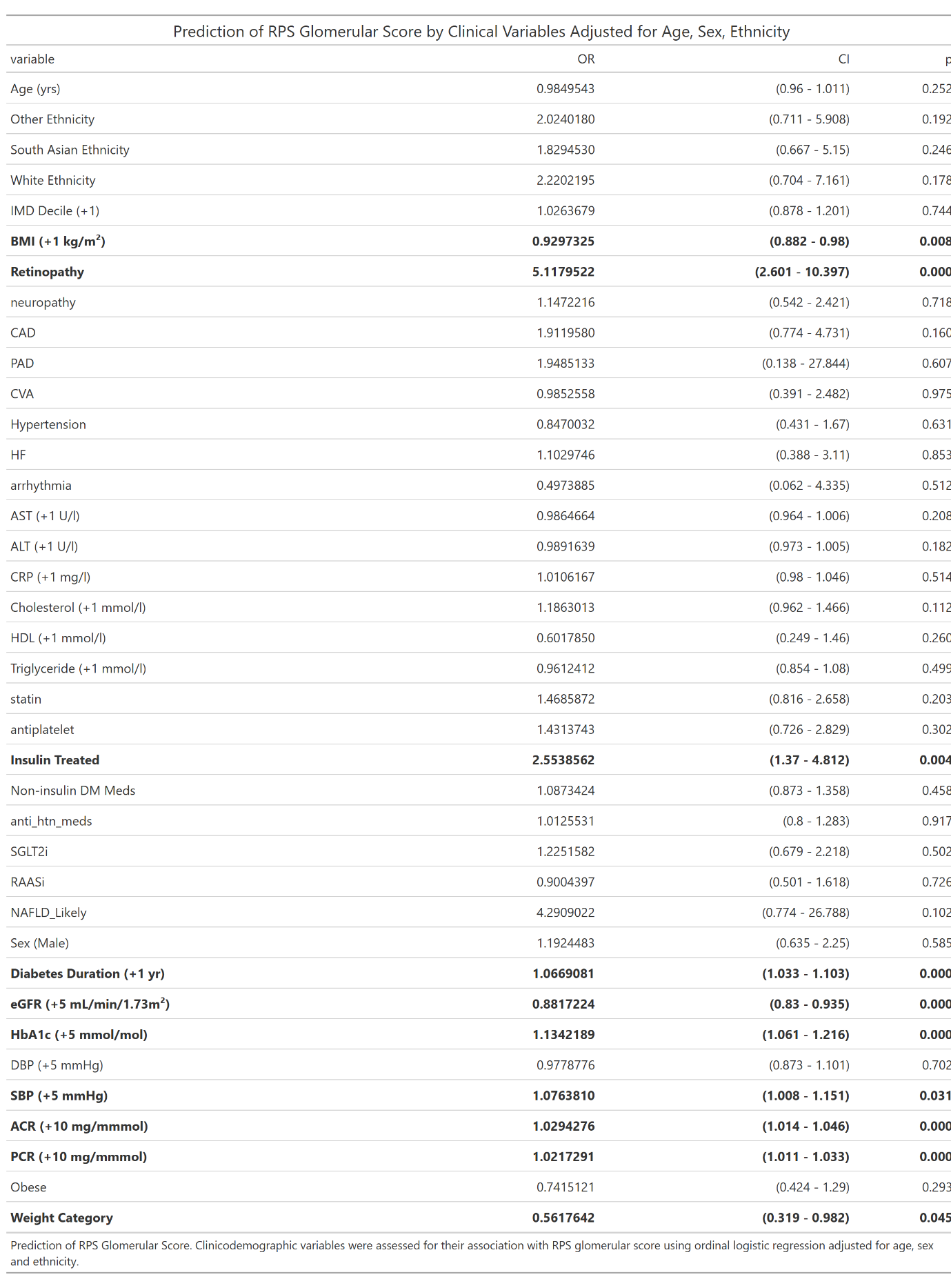


Age, Sex and Ethnicity Adjusted Prediction of RPS Glomerular Score by Clinical Variables without imputation. Clinicodemographic variables were assessed for their association with RPS glomerular score using ordinal logistic regression adjusted for age, sex and ethnicity.

*Analyses without Imputation: PCA and Cluster Analysis*


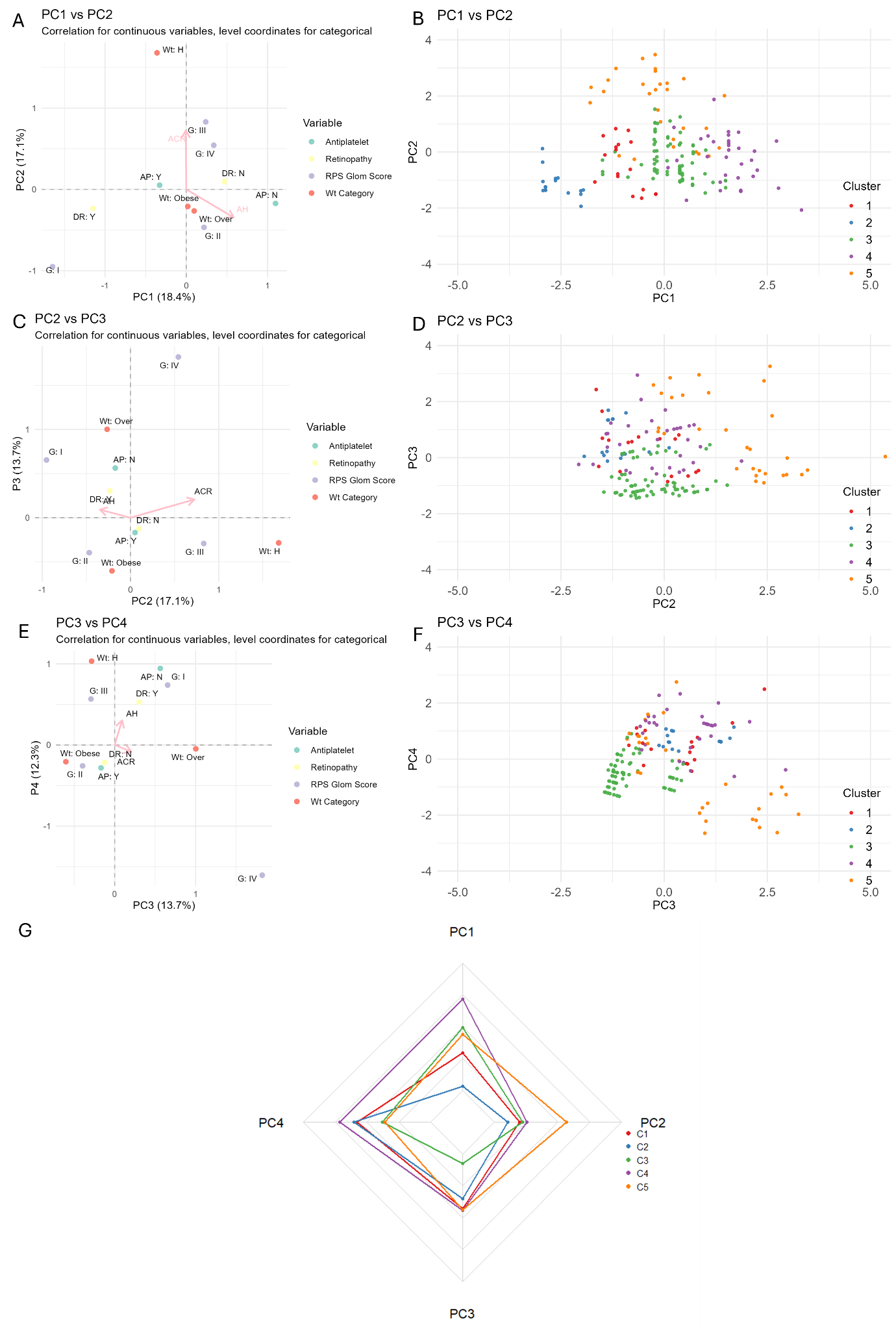


Figure 2: Cluster analysis. PCA for mixed data was performed on selected variables. The level co-ordinates for categorical variables are shown with correlation vectors of continuous traits overlaid for PC1 vs PC2 (A), PC2 vs PC3 (C), PC3 vs PC4 (E). Ensemble clustering was performed on the 4 PCs which explained > 60% of variation. B, D and F show the individual coordinates for each participant in PC1 vs PC2 (B), PC2 vs PC3 (D), PC3 vs PC4 (F), with cluster assignment depicted by colour. (G) shows a radar plot of median PC scores for clusters 1 - 5. G = glomerular score, AH = anti-hypertensives, AP = antiplatelet use, DR = retinopathy, Wt: H = healthy weight, Wt: Over = overweight; Wt: Obese = obese.


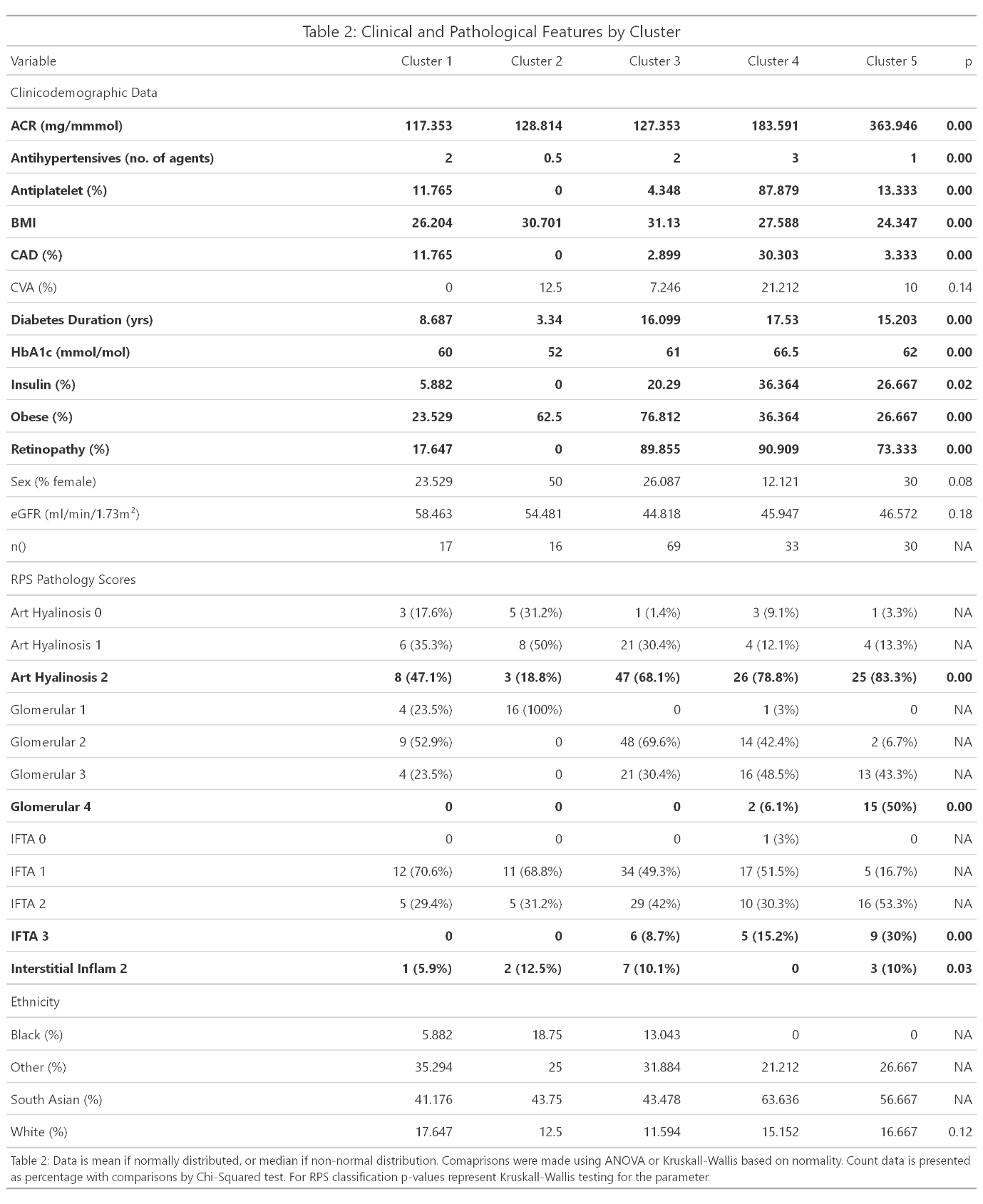


Comparison of clusters. Data is presented as median or count percentage. Comparisons were made using ANOVA or Kruskall-Wallis based on normality or for count data comparisons are made by Chi-Squared test. For RPS classification p-values represent Kruskall-Wallis testing for the parameter.
