# Supplementary material for: Histological and clinical phenotypes of diabetic kidney disease: a baseline analysis of the HEROIC study
Source: Clin Kidney J. 2026 Jun 25;19(7):sfag215. doi: 10.1093/ckj/sfag215 (PMC13373966; doi:10.1093/ckj/sfag215)
Supplement: sfag215_Supplemental_Files [file sfag215_supplemental_files.zip › Supplementary_Methods_Resubmission.docx]

Additional Methods

*Additional Patient Selection:*

Moderate progression risk was defined as either a rapidly falling eGFR (>5mL/min/1.73m²/year decline in eGFR measured by three or more samples over at least 6 months) or significant albuminuria (ACR > 30 mg/mmol); high risk was defined as the presence of both. Patients could also be recruited if a clinically indicated biopsy which demonstrated DKD without significant non-diabetic renal disease. Patients were excluded if they were unable to give informed consent, had contra-indications to MRI or renal biopsy, a life expectancy under 3 years in the judgment of PI, were pregnant, or had an alternative renal diagnosis which was likely to contribute towards renal progression.

*Additional Clinical Data Synthesis Methods:*

Where data was missing from baseline visit, past medical history was enriched from their hospital electronic health records (EHR) and, where available, primary care summary. Routine blood tests were performed including renal, bone, lipid and liver profiles, bicarbonate, c-reactive protein (CRP), haemoglobin A1c, and urine creatinine, protein and albumin. Missing laboratory or clinical variables were taken from outpatient appointments within 6 months of biopsy. Where multiple laboratory samples existed on the testing date, the first sample was used. mGFR was assessed by ^99m^Tc-DTPA plasma clearance.

Diabetes type was recorded from EHR and date of onset from either EHR or patient history. Coronary artery disease was recorded if there was record of myocardial infarction, ischaemic heart disease, angina or angiographic data. Peripheral vascular disease synonyms included limb ischaemia, peripheral embolic events, aortic aneurism disease. Cerebrovascular disease referred to any ischaemic or haemorrhagic stroke along with transient ischaemic attack. Heart failure referred to any diagnosis of congestive cardiac failure or heart failure (including preserved ejection fraction). Retinopathy date was the first known diagnosis of diabetic retinopathy or maculopathy.

Obesity was defined and staged depending on ethnicity in line with UK guidelines(1) into non-overweight, overweight, obese. For summary data a dichotomous obese vs non-obese state is shown but for regression analyses and cluster analyses it was treated as a factor (ordered in the case of regression and non-ordered since this ordered data is not handled by the R package PCAmixdata(2)).

*Data Preparation and Presentation*

Data distribution was tested by Shapiro tests and data are presented as mean (+/- SD) or median (IQR) as appropriate for distribution. Where data has been stratified by ethnicity comparisons are made using ANOVA or Kruskall-Wallis tests based on normality. For PCA and cluster analyses variables were screened for completeness (excluding variables with >20% missingness) and homogeneity (removing variables > 95% homogeneity).

*Handling Data Outside Limits of Detection*

Where laboratory samples fell below the lower limit of detection (LoD) threshold, they were treated as half the value of the LoD(3). Only albumin and protein were recorded above limits of detection. For albumin:creatinine and protein:creatinine ratio (ACR/PCR) where albumin or protein fell above the LoD, the albumin/protein LoD was used to calculate the ACR or PCR.

*Handling Missing Data*

If possible ACR was calculated from PCR using established methods(4). Multivariate imputation by chained equations (MICE R package(5)) was employed to impute missing data for regression analyses. M = 10 imputed datasets were generated using predictive mean matching. Distribution of imputed datasets were averaged and the distribution of this mean imputed dataset compared to observed data for continuous variables which had missing data. PCA for mix data is not currently compatible with MICE therefore imputation using factor analysis of mixed data (missMDA package(6)) was employed since this is designed as preparatory stage prior to PCA of mixed data. The optimal number of dimensions for imputeFAMD was determined by the estim_ncpFAMD function by cross-validation. Distribution of imputed, complete data were compared to raw, observed data for cluster variables.

*Ensemble Consensus Clustering*

Clustering was performed on variables related to the hypothesis that there may be clusters of patients with classic nodular sclerosis and other populations with alternative forms of glomerular pathology related to metabolic syndrome or vasculopathy. The following variables were selected to reflect markers of glomerular disease and of metabolic/cardiovascular disease:

- Markers of glomerular disease
  - ACR
  - RPS glomerular score
- Markers of metabolic and cardiovascular disease:
  - Weight category: since the relationship between BMI and cardiovascular risk varies by ethnicity, we have used ethnicity adjusted weight categories as outlined by NICE(1)
  - Number of antihypertensives was selected as a marker for the severity of hypertension
  - Antiplatelet use: it is noted that our cohort has lower rates of cardiovascular disease than other cohorts and to minimise the impact of recall bias and poor coding impacting the clustering we sought to use a proxy for atherosclerotic disease, therefore we have utilised antiplatelet prescriptions(7).
  - Retinopathy was used as a marker of existing microvascular disease and cumulative glycaemic exposure. There is a national screening programme for retinopathy in the UK and it is felt to be reasonably well coded(8).

Consensus clustering was performed using the diceR package(9) on the first n PCs which explained > 60% of cumulative variance. The following clustering algorithms were used: k-means, hierarchical clustering and partition around mediod clustering with consensus determine by k-mediod method. 50 resampling iterations were used with proportion used in each sampling set as 0.8 (p.item default). Optimal K was determined by diceR’s internal stability evaluation, with K chosen to maximise cluster stability, though K was constrained to between 2 and 5 to optimise interpretability.

*eGFR equation validation*

To illustrate clinical impact, we estimated the proportions of patients with misclassified KDIGO CKD grades and the ability of each equation to improve classification (net reclassification index, NRI). Creatinine and mGFR measurements were not always taken simultaneously, therefore, drift in GFR was corrected by linear regression and metrics presented are adjusted for the interval between eGFR and mGFR.

For analysis of mGFR-eGFR performance, due to covid restrictions, some participants first mGFR was at their year 1 visit, in which case this and the year 1 creatinine value was used. Given the interval between mGFR and creatinine measurement varied the following equations were used to adjust for this interval:

$$Interval={Date}_{Creatinine}- {Date}_{mGFR}$$

$$Bias(adjusted)=mGFR-{eGFR}_{Raw}-(\beta\cdot Interval)$$

$${eGFR}_{adjusted}={eGFR}_{raw}-(\beta.Interval)$$

Reported metrics are based on adjusted bias and adjusted eGFR.

1. Overweight and obesity management. 2025.

2. Marie Chavent VK, Amaury Labenne, Benoit Liquet and Jerome Saracco. PCAmixdata: Multivariate Analysis of Mixed Data. 2017.

3. Barescut J, Lariviere D, Stocki T, Wood MD, Beresford NA, Copplestone D. Limit of detection values in data analysis: Do they matter? | Radioprotection | Cambridge Core. Radioprotection. 2011/01;46(6).

4. K S, GN N, ME G, Y S, SH B, J C, et al. Conversion of Urine Protein-Creatinine Ratio or Urine Dipstick Protein to Urine Albumin-Creatinine Ratio for Use in Chronic Kidney Disease Screening and Prognosis : An Individual Participant-Based Meta-analysis - PubMed. Annals of internal medicine. 09/15/2020;173(6).

5. Buuren Sv, Groothuis-Oudshoorn K. mice: Multivariate Imputation by Chained Equations in R. Journal of Statistical Software. 2011;45(3).

6. Josse J, Husson F. missMDA: A Package for Handling Missing Values in Multivariate Data Analysis. Journal of Statistical Software. 2016;70(1):1–31.

7. Pouwels KB, Voorham J, Hak E, Denig P, Pouwels KB, Voorham J, et al. Identification of major cardiovascular events in patients with diabetes using primary care data. BMC Health Services Research 2016 16:1. 2016–04–02;16(1).

8. Martín-Merino E, Fortuny J, Rivero E, García-Rodríguez LA. Validation of Diabetic Retinopathy and Maculopathy Diagnoses Recorded in a U.K. Primary Care Database. Diabetes Care. 2012 Mar 13;35(4).

9. Talhouk DCaA. diceR: Diverse Cluster Ensemble in R. 2025.
