# Supplementary material for: Histological and clinical phenotypes of diabetic kidney disease: a baseline analysis of the HEROIC study
Source: Clin Kidney J. 2026 Jun 25;19(7):sfag215. doi: 10.1093/ckj/sfag215 (PMC13373966; doi:10.1093/ckj/sfag215)
Supplement: sfag215_Supplemental_Files [file sfag215_supplemental_files.zip › Supp_Figs_V3.docx]

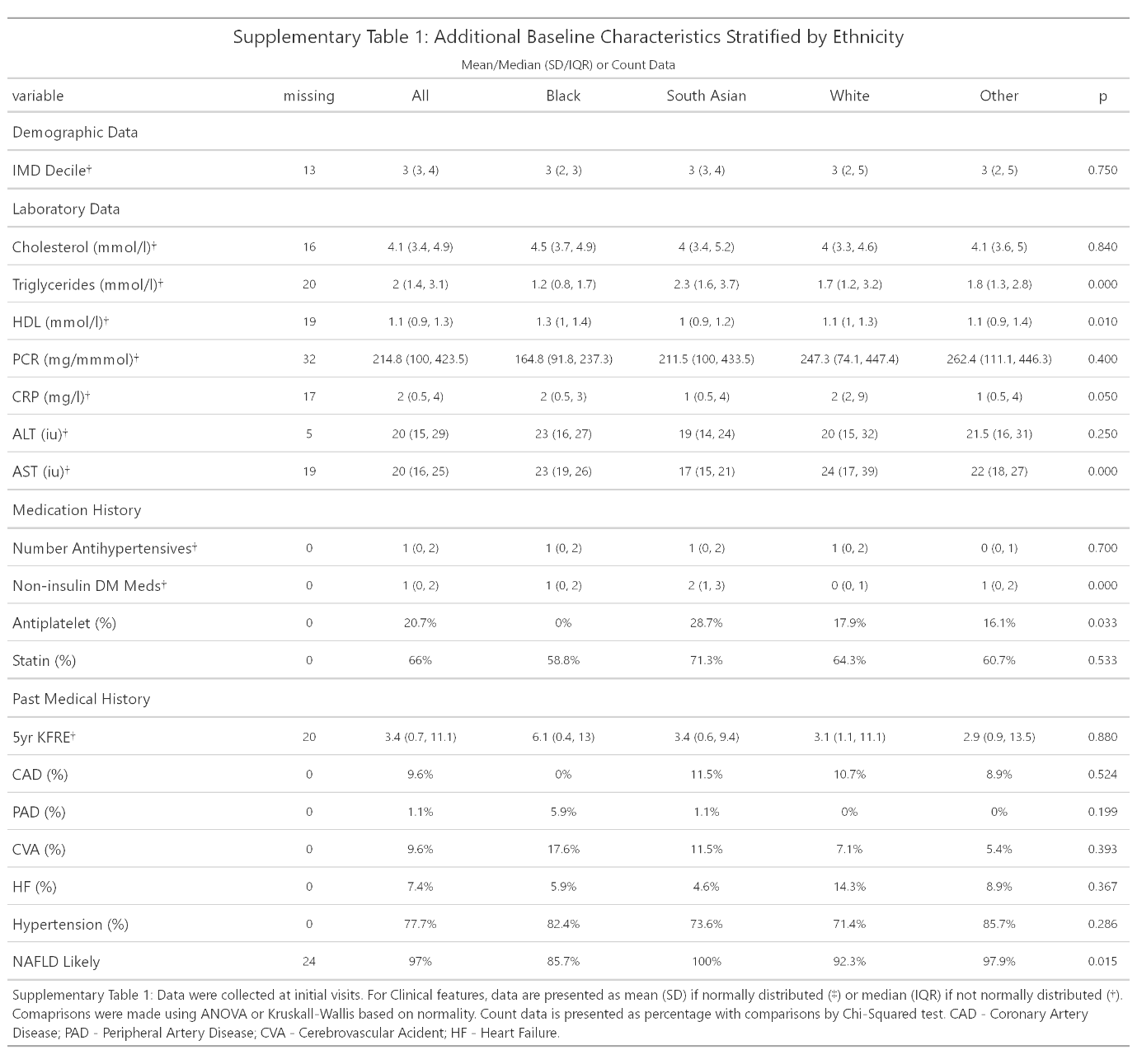


Supplementary Table 1: Data were collected at initial visits. For Clinical features, data are presented as mean (SD) if normally distributed (‡) or median (IQR) if not normally distributed (†). Comparisons were made using ANOVA or Kruskall-Wallis based on normality. Count data is presented as percentage with comparisons by Chi-Squared test. CAD - Coronary Artery Disease; PAD - Peripheral Artery Disease; CVA - Cerebrovascular Accident; HF - Heart Failure.


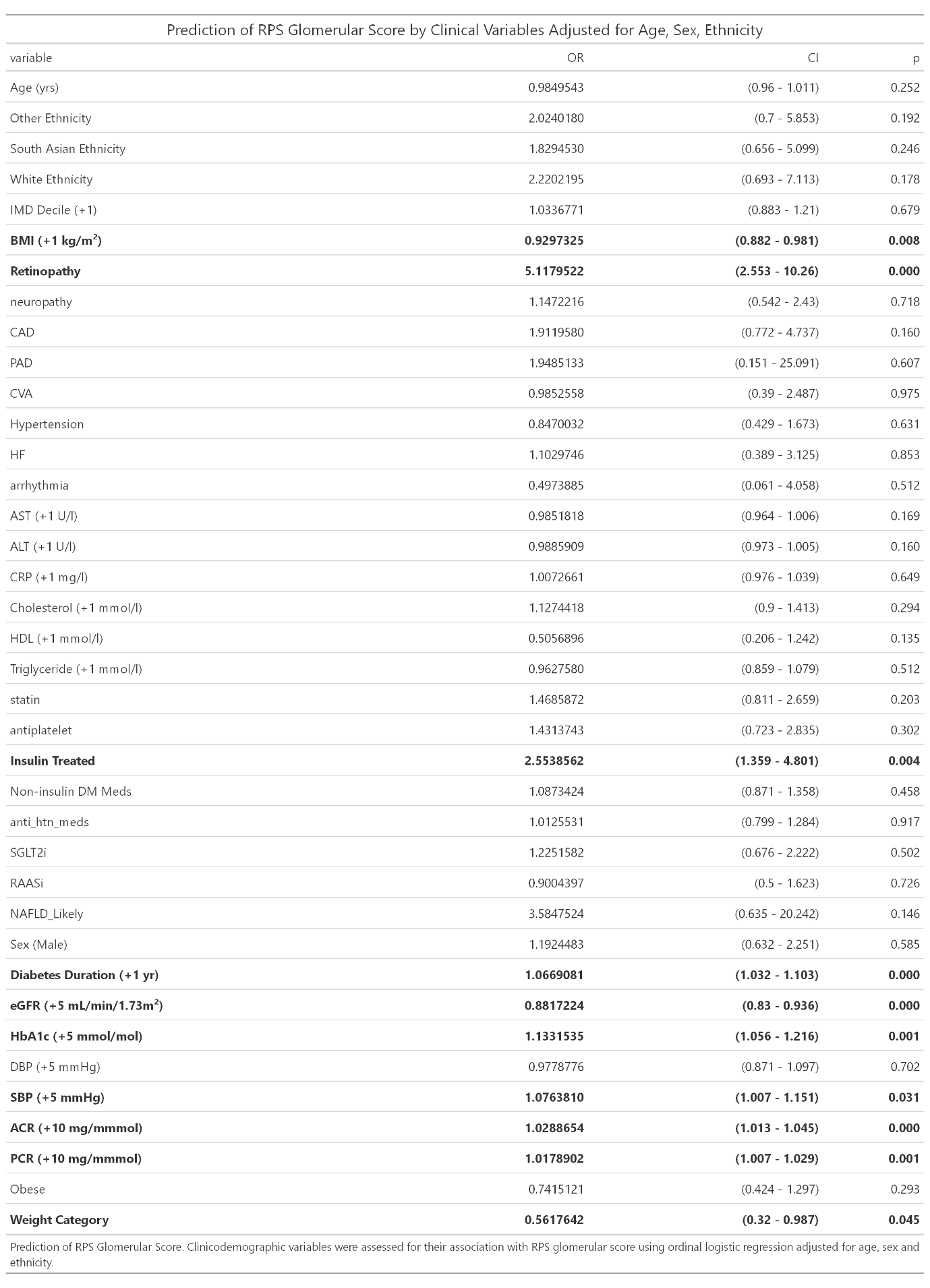


Supplementary Table 2: Age, Sex and Ethnicity Adjusted Prediction of RPS Glomerular Score by Clinical Variables. Clinicodemographic variables were assessed for their association with RPS glomerular score using ordinal logistic regression adjusted for age, sex and ethnicity.


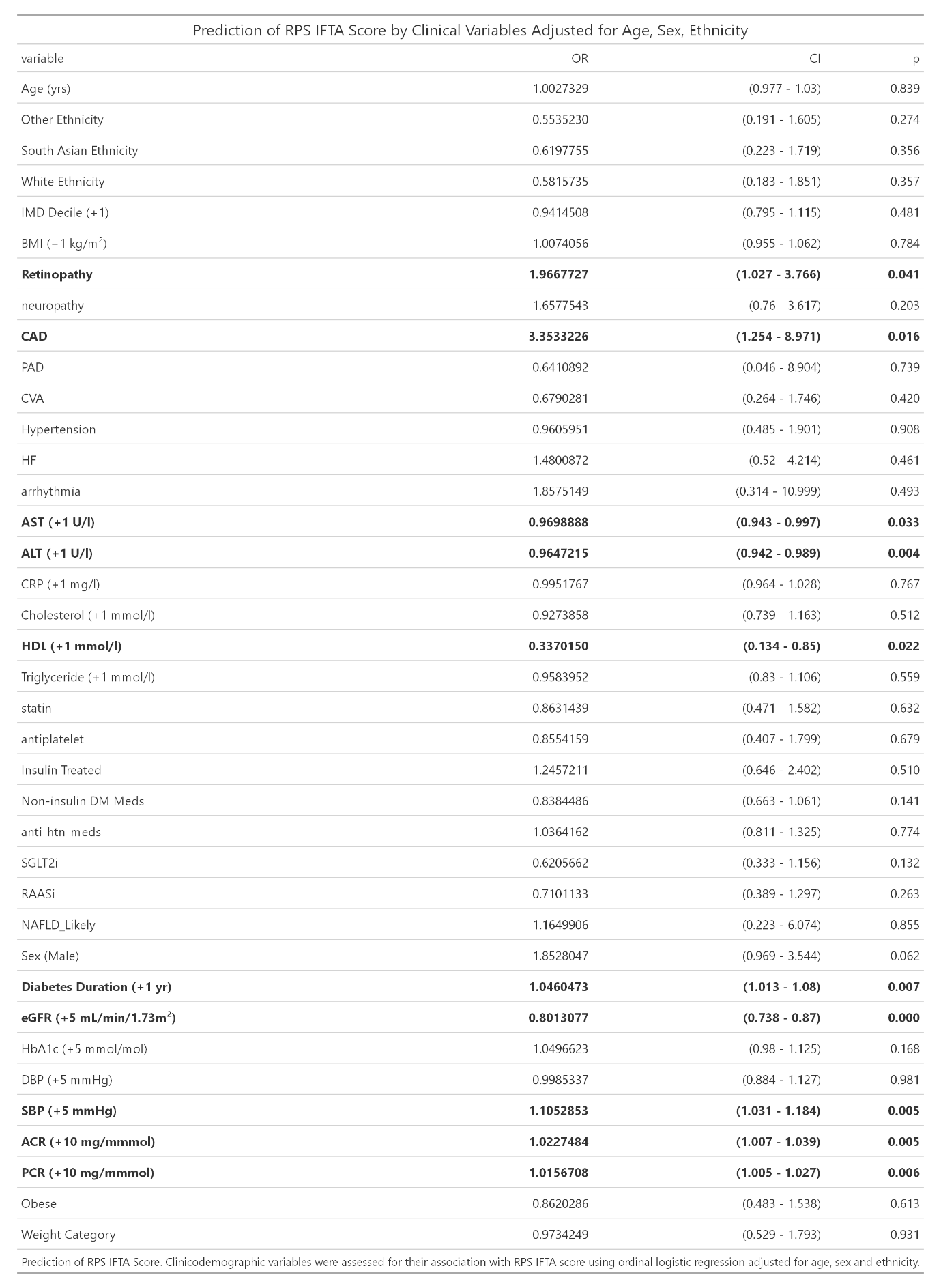


Supplementary Table 3: Age, Sex and Ethnicity Adjusted Prediction of RPS IFTA Score by Clinical Variables. Clinicodemographic variables were assessed for their association with RPS IFTA score using ordinal logistic regression adjusted for age, sex and ethnicity.


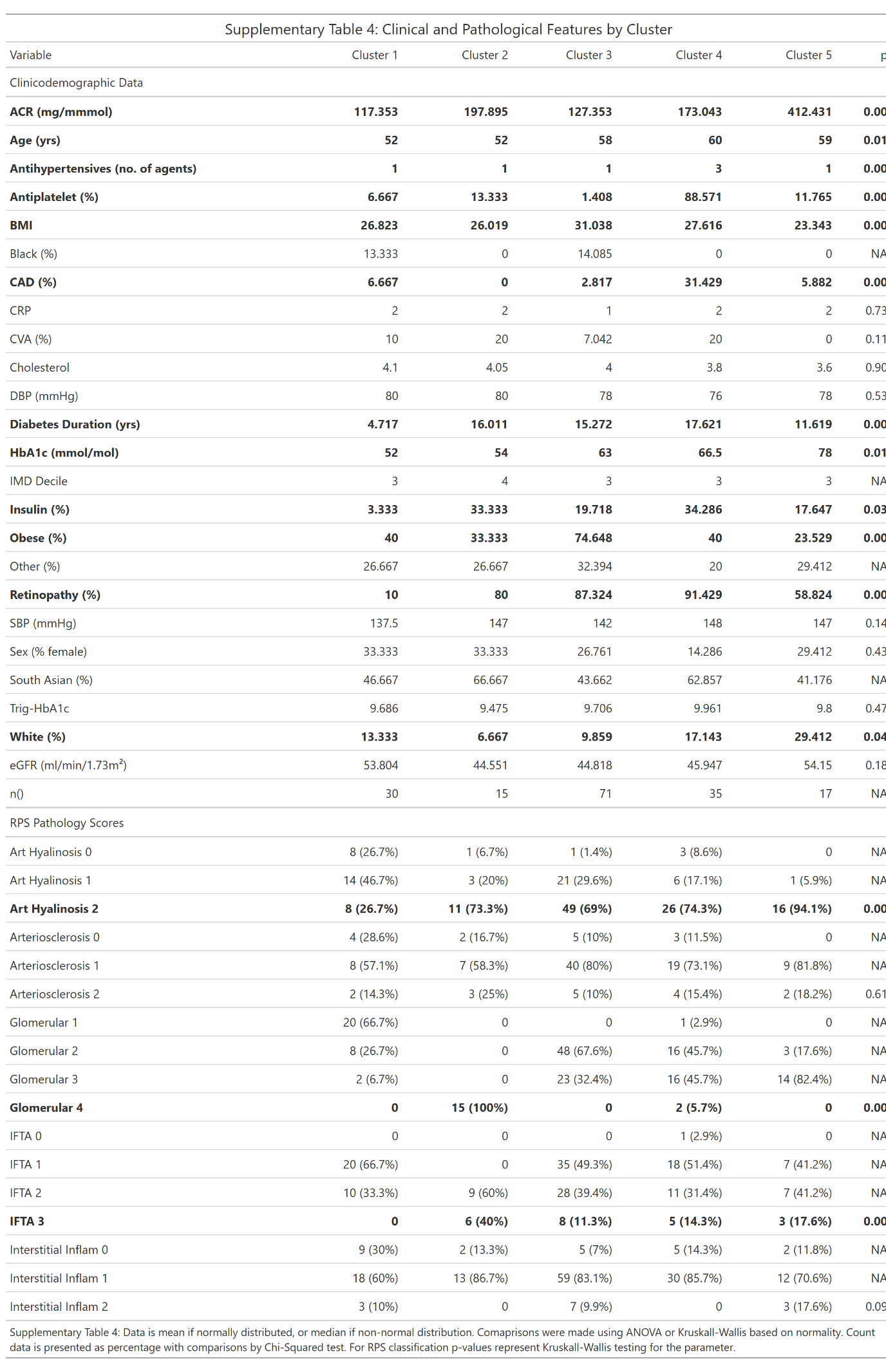


Supplementary Table 4: Extended comparison of clusters. Data is mean if normally distributed, or median if non-normal distribution. Comparisons were made using ANOVA or Kruskall-Wallis based on normality. Count data is presented as percentage with comparisons by Chi-Squared test. For RPS classification p-values represent Kruskall-Wallis testing for the parameter.


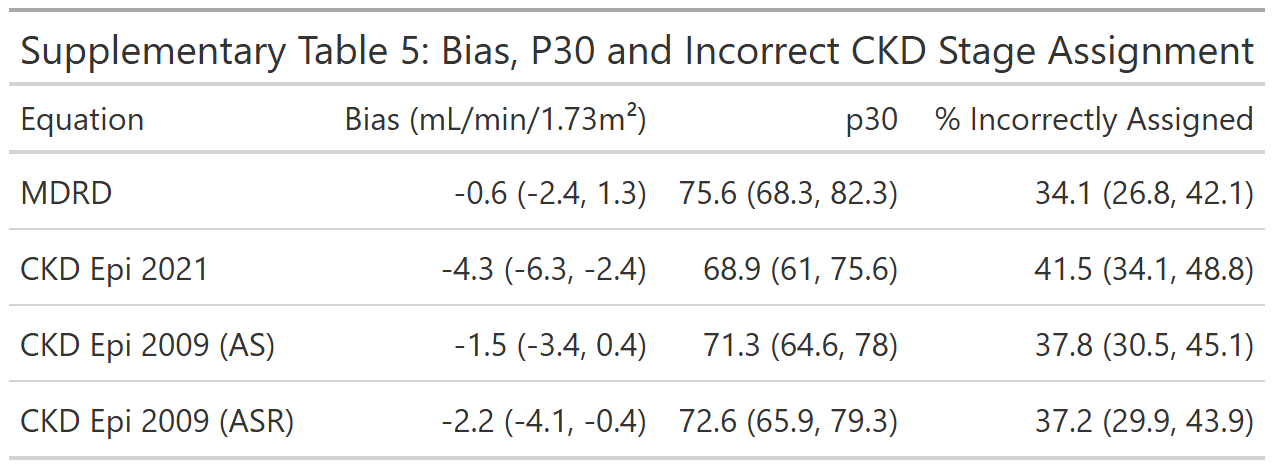


Supplementary Table 5: Bias, P30, and Percent Incorrectly Assigned CKD Stage. Bias (mGFR – eGFR) has been adjusted for the interval between mGFR and eGFR testing. P30 represents the percentage of participants for whom the interval-adjusted eGFR was within 30% of mGFR. Percent incorrectly assigned CKD stage reflects the proportion of participants whose interval-adjusted eGFR CKD stage differed from the true stage defined by mGFR. For CKD-EPI 2009, results are shown for the base equation using age and sex [CKD-EPI 2009 (AS)] and with the addition of the Black race coefficient [CKD-EPI 2009 (ASR)]. Data are presented with 95% confidence intervals.


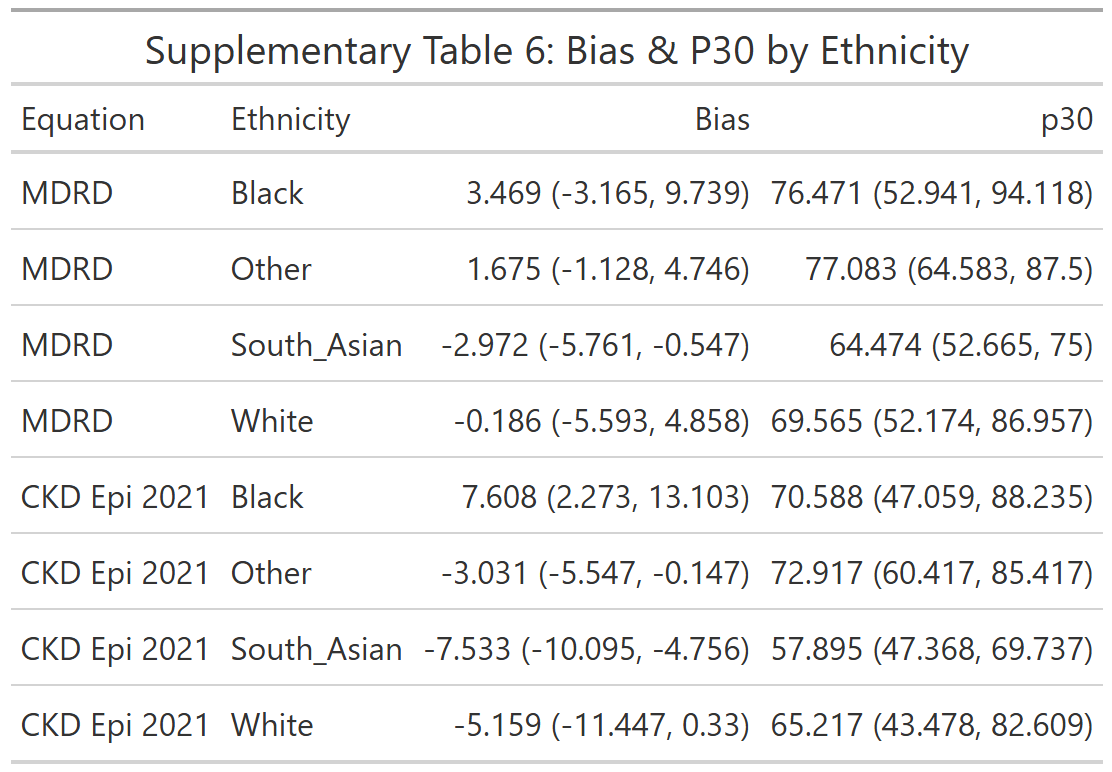


Supplementary Table 6: Bias and P30 stratified by ethnicity. Bias (mGFR – eGFR) has been adjusted for the interval between mGFR and eGFR testing. P30 represents the percentage of participants for whom the interval-adjusted eGFR was within 30% of mGFR. Data are presented with 95% confidence intervals.


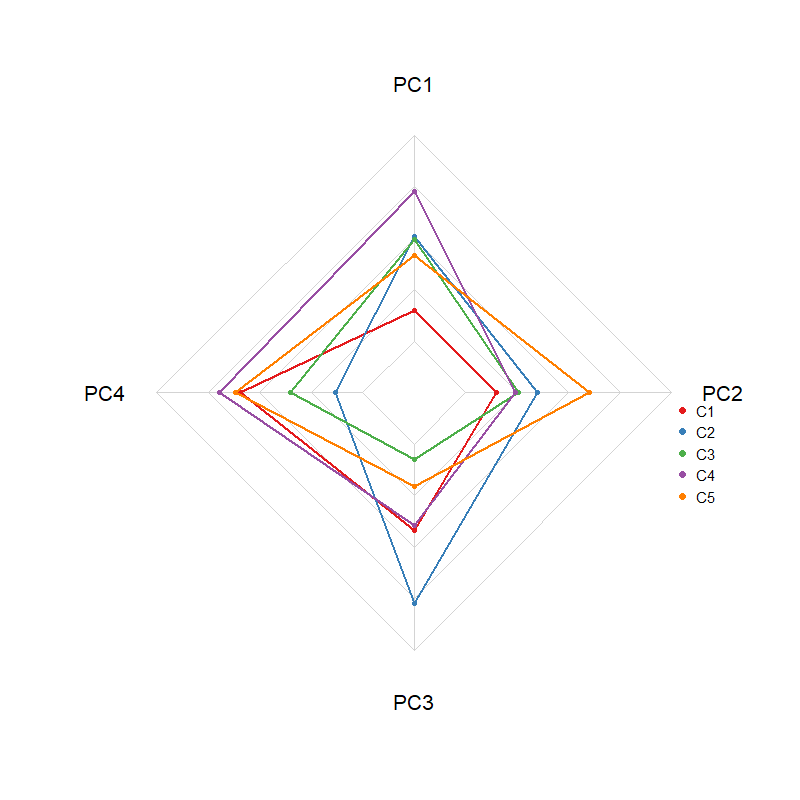


Supplementary Figure 1: PCA of mixed data was performed on selected variables with 4 principal components explaining > 60% of variance. Ensemble consensus clustering was performed on individual participants coordinates in PC space. The median score for each cluster per principal component is shown in a radar chart.
